# Inhibiting TRK Proteins in Clinical Cancer Therapy

**DOI:** 10.3390/cancers10040105

**Published:** 2018-04-04

**Authors:** Allison M. Lange, Hui-Wen Lo

**Affiliations:** 1 Department of Cancer Biology, Wake Forest University School of Medicine, Winston-Salem, NC 27157, USA; amlange9@gmail.com; 2 Comprehensive Cancer Center, Wake Forest University School of Medicine, Winston-Salem, NC 27157, USA

**Keywords:** NTRK gene fusions, TRKA, TRKB, TRKC, tyrosine kinase inhibitor, cancer, neurotrophin

## Abstract

Gene rearrangements resulting in the aberrant activity of tyrosine kinases have been identified as drivers of oncogenesis in a variety of cancers. The tropomyosin receptor kinase (TRK) family of tyrosine receptor kinases is emerging as an important target for cancer therapeutics. The TRK family contains three members, TRKA, TRKB, and TRKC, and these proteins are encoded by the genes NTRK1, NTRK2, and NTRK3, respectively. To activate TRK receptors, neurotrophins bind to the extracellular region stimulating dimerization, phosphorylation, and activation of downstream signaling pathways. Major known downstream pathways include RAS/MAPK/ERK, PLCγ, and PI3K/Akt. While being rare in most cancers, TRK fusions with other proteins have been well-established as oncogenic events in specific malignancies, including glioblastoma, papillary thyroid carcinoma, and secretory breast carcinomas. TRK protein amplification as well as alternative splicing events have also been described as contributors to cancer pathogenesis. For patients harboring alterations in TRK expression or activity, TRK inhibition emerges as an important therapeutic target. To date, multiple trials testing TRK-inhibiting compounds in various cancers are underway. In this review, we will summarize the current therapeutic trials for neoplasms involving NTKR gene alterations, as well as the promises and setbacks that are associated with targeting gene fusions.

## 1. Introduction

Mutations or rearrangements in the tropomyosin receptor kinase (TRK) family of receptor tyrosine kinases has recently been described as a mechanism of oncogenesis. The discovery of small molecule TRK inhibitors has generated much excitement in the cancer research community, and early clinical analysis of several compounds has yielded promising results for a variety of cancers. Understanding this family of tyrosine kinase receptors, as well as the related kinase receptor proteins, is essential to developing effective treatments that are targeting gene rearrangements. 

The TRK family of receptor tyrosine kinases consists of three members, TRKA, TRKB, and TRKC. These TRK receptors contribute to neuronal development, function, survival, and proliferation during the development and into adulthood [1,2]. Like other receptor tyrosine kinases, TRK proteins are activated following ligand binding to the extracellular domain of the receptor. Ligands for TRK proteins are the neurotrophins, a family of closely-related secreted proteins that were discovered and classified initially as proteins that were able to promote differentiation and survival in both sympathetic and sensory nerves [2,3]. Signaling via TRK proteins is achieved with specific neurotrophin ligands, which triggers receptor dimerization and phosphorylation, leading to the activation of downstream signaling cascades. Each TRK receptor is activated by a distinct neurotrophin. Nerve growth factor (NGF) stimulates TRKA; brain-derived growth factor (BDGF) and neurotrpphins-4/5 activate TRKB whileneurotrpphins-3 activates TRKC. Structurally, TKR proteins share a similar design with an extracellular ligand binding domain, a transmembrane domain, and an intracellular kinase domain [4,5]. Through binding to TRK receptors, neorotrophinss activate Ras, phosphatidylinositol-3 (PI3)-kinase, and phospholipase C-γ1 signaling pathways [2]. By this signaling, neurotrophins ultimately regulate cell fate decisions, axon growth, dendrite growth, and pruning. They also have additional subtler roles unrelated to the nervous system. In recent years, three rare human genetic disorders, which resulted in deleterious effects on sensory perception, cognition, and a variety of behaviors, have been shown to be attributable to mutations in BDNF and two of the TRK receptors [6,7]. In addition to the expression in the central nervous system (CNS), TRK proteins have also been identified in non-neuronal normal tissues and cells, including pancreatic beta cells, monocytes, lungs, and bone [8].

TRKA is the founding member and best studied of the TRK family of proteins. TRKA is activated by NGF, which has a well-studied role in the mediation of pain, itch, and inflammation, and the development and maintenance of cholinergic, sympathetic, and sensory neurons [9]. Following NGF binding, TRKA activates cell proliferation pathways, including the Ras/MAPK/ERK and the PLCγ/PI3K pathways. Mutations interfering with the function of TRKA in humans have been reported to cause congenital insensitivity to pain with anhidrosis syndrome, as well as, mental retardation and self-mutilating behavior [10,11]. Physiologically, TRKA is expressed in both the developing and adult nervous system where it controls the differentiation and survival of neurons [12]. In colorectal cancer patients, chromosomal rearrangements involving the NTRK1 gene (encoding the TRKA protein) are shown in a small subset of patients and are associated with the constitutive activation of the kinase domain of TRKA. In turn, activated TRKA-fusion proteins are associated with the proliferation and survival in colorectal cancer tumors. 

TRKB, encoded by NTRK2, is activated by BDNF and has been shown to promote neural cell plasticity and survival [13]. In humans, TRKB expression is highest in the prefrontal cortex, amygdala, and the occipital lobe [14]. Because of its function and expression, altered TRKB expression is implicated in CNS pathologies. Importantly, reduced TRKB protein is observed in post-mortem samples of Alzheimer’s disease patients [6,15,16], as well as, Parkinson’s disease [17], demonstrating the importance of TRKB to neuronal survival. Decreased TRKB expression is also observed in patients suffering from major depressive disorder [18] and in the post-mortem analysis of the prefrontal cortex of suicide victims and schizophrenics [7,19,20,21], indicating that TRKB also has a role in mood stabilization and synaptic signaling. As a treatment target, inhibiting TRKB may have undesirable side effects including ataxia, anhedonia, lethargy, and depression [11]. 

Similar to TRKA and TRKB, TRKC also promotes neuronal differentiation and survival. Following neurotrophin-3 stimulation TRKC, apoptosis is prevented through the activation of the PI3K/AKT pathway. Mutations in TRKC resulting in an inactive protein have been identified as a cause for the Hirschsprung disease, resulting in the absence of nerve cells in the myenteric and submucosal plexus and causing gastrointestinal complications [22]. TRKC, along with TRKA and TRKB have a recently recognized role in cancer signaling, which is discussed below.

## 2. TRK Signaling in Cancer

While the TRK family of receptors play an essential role in neuronal growth and development, the rearranged state may be a driving force for malignancies. Gene fusions lead to chimeric TRK proteins that either lead to the overexpression of the kinase domain or possess constitutive activity of the kinase function. This rearranged state, either through point mutations, chromosome rearrangements and gene fusions, or deletions, results in the spontaneous ligand-independent dimerization and subsequent activation of signal transduction [23]. 

Fusions of NTRK proteins have been identified in various solid tumors, including the lung, gastrointestinal tract, thyroid, primary brain tumors, and sarcomas [4]. Besides solid tumors, there have also been reports of hematological malignancies that were driven by NTRK fusions, including acute myeloid leukemia (AML) [24]. While NTRK fusion expression in all cancers is rare, it is the defining oncogenic driver and a diagnostic marker in some cancers, including secretory breast carcinoma, congenital infantile fibrosarcoma, and mammary analogue secretory carcinoma [1,5]. The identification of recurrent NTRK gene rearrangements, along with preclinical data demonstrating that these oncogenes drive tumorigenesis has initiated the clinical examination of kinase inhibitors as therapeutic targets. To date, all the known mechanisms of oncogenic TRKA activation (fusions, deletions, and alternative splicing) involve some truncation of the extracellular domain, suggesting the existence of a common regulatory domain in this extracellular region [25]. 

Besides protein fusions, the neurotrophins themselves have been hypothesized to promote tumor growth, and *in vitro* studies with tumor lines have demonstrated this capability. Singer et al. showed that NGF is capable of stimulating the proliferation of several glioblastoma cell lines, as well as stimulating the further secretion of NGF [9]. Additionally, NGF has demonstrated a capability for progression of non-neuronal tumors, including pancreatic [26], prostatic [27], lung [28], ovarian [29], and medullary thyroid [30]. 

## 3. TRK Fusion Oncoproteins

Recognition of gene fusions as drivers for oncogenesis began with the identification of BCR-Abl as an initiator for chronic myelogenous leukemia in 1982 [31]. Since this discovery, fusion genes of kinases have been additionally identified in solid tumors, including non-small cell lung cancer (NSCLC) [32], prostate cancer [33], glioblastoma [34], and lung adenocarcinoma [35]. With advances in massively parallel sequencing of the cancer genome, as well as, the availability of large scale sequencing data, the identification of gene fusions has become more straightforward and more reliable [36]. Regarding NTRK and cancer, gene fusions represent the primary molecular alteration that confers oncogenic behavior. Across all the known gene fusions of TRK proteins, the 3′ region of the NTRK gene is fused with the 5′ region of its fusion partner, and the resulting chimeric protein is then either overexpressed or constitutively active [4] (Table 1).

NTRK fusions were originally identified in 1986 in colon cancer when a TPM3-NTRK1 translocation was detected in a tumor biopsy [37]. Since this observation, gene fusions involving NTRK1, 2, and 3 genes have been documented in 11 specific tumor types, most notably NSCLC, papillary thyroid carcinoma [38], secretory breast cancer [39], and glioblastoma [40,41]. Several NTRK gene fusions have been identified as potential initiators of tumorigenesis, and are present in the majority of certain subtypes. For example, 100% of all mammary analogue secretory carcinomas (MASC) and 93% of secretory breast cancers exhibit the ETV6-NTRK3 gene fusion [42]. Interestingly, a recent study reported the identification and the functional characterization of a new SCYL3-NTRK1 fusion gene in a patient with colorectal cancer, which is oncogenic and sensitive to TRKA inhibitors [43]. As sequencing techniques continue to develop, it is expected that more NTRK gene fusions will be identified, renewing interest in TRK proteins as therapeutic targets. 

## 4. Alternative Splicing and Overexpression of TRK Proteins

Other molecular alterations to TRK have been reported, but with a lower penetrance. An in-frame deletion of NTRK1 in de novo AML has been reported, but this appears to be sporadic and rare [77]. Likewise, a TRKA alternative splicing event has been observed in neuroblastoma [23]. While being relatively specific to neuroblastoma cells, this splice variant was shown to promote angiogenesis and a stress-resistant phenotype [23]. Interestingly, Tacconelli et al. showed that this splice variant had spontaneous tyrosine kinase activity, contributing to higher PI3K and NFkB activity [23]. With increased sensitivity of alternative splicing detection methodswe expect to see more splice variants identified in the future.

Since NTRK overexpression contributes to tumorigenesis and progression, cancers with aberrant NTRK signaling may benefit from TRK inhibitors. In 2009, Lagadec et al. showed that breast tumors expressed higher levels of TRKA and phospho-TRKA when compared to normal breast tissue [3]. Furthermore, they demonstrated in vitro that TRKA overexpression promoted proliferation, migration, and invasion, and that PI3K-Akt and ERK/MAPK pathways were activated by TRKA to promote this aggressive phenotype. These results were recapitulated in animal models, where xenografted mice with TRKA overexpression had accelerated tumor growth, angiogenesis, and metastasis [3]. 

Other isolated studies have recognized overexpression of TRKA in neuroblastoma [78] and lung cancer [79]. In a study probing 381 cDNA samples from 22 unique cancers, Narayanan et al. showed 50–1000-fold overexpression of TRKA in 100% of pheochromocytoma cases, as compared to normal tissue. In another study, TRKA was observed to be overexpressed in cancers of the pancreas (5/17), ovary (7/20), and esophagus (9/20) [80]. Like TRKA, TRKC was also shown to have activating mutations in the breast, lung, colon, and pancreatic cancers. Gene fusions, as well as the overexpression of NTRK3, have also been identified as potential contributors to certain cancers, including medulloblastoma, neuroblastoma, and melanoma [22]. Because NTRK alterations occur in a variety of cancer histologies, investigation and investments in TRK inhibitors may be proven to be a beneficial and revolutionary treatment for these malignancies.

## 5. Targeting TRK in Cancer

Expression of TRKA protein in cancer may confer a more favorable prognosis in certain neoplasms. For example, TRKA expression in neuroblastoma confers a good prognosis via a potential tumor-suppressing role for TRKA and NGF signaling in neuroblastoma cells, resulting in differentiation, growth arrest, and angiogenesis inhibition [78,81,82,83,84]. Another study found that higher TRKA expression in neuroblastomas was associated with favorable clinical features, while expression and signaling via TRKC corresponded to a more aggressive and invasive neuroblastoma phenotype [85]. This favorable prognosis may only be relevant for TRKA, and TRKC and TRKB are linked with more aggressive clinical features. TRKB is implicated in the activation of autocrine and paracrine signaling in tumor cells, facilitating angiogenesis and resistance to anticancer drugs [83]. This phenotype has been observed in neuroblastoma, although it is predicted to have relevance across other tumor types [86,87]. Furthermore, expression of NTRK1 rearrangements in papillary thyroid cancer also confers a worse prognosis when compared to patients without this fusion gene [88]. However, the introduction and refining of kinase inhibitor therapies, combined with a defined oncogenic target, and the extremely limited appearance of clinical resistance mechanisms, may prove to make NTRK gene alteration cancers may be easier to control. 

## 6. Clinical Trials Involving TRK Inhibitors

Identification of fusion genes altering the activity of NTRK kinase domains has prompted interest in developing tyrosine kinase inhibitors specific for these proteins. Interestingly, the kinase domain of TRK proteins is remarkably conserved. TRKB and TRKC share 100% identical residues in the ATP binding sites, and there is only a 2 of 40 residue deviation between TRKA and TRKB [89]. Because of the remarkable conservation, efforts to develop a pan-TRK inhibitor seem more feasible than generating an inhibitor with specificity. For the cancer treatment paradigm, generating a pan-TRK inhibitor translates to broader antitumor activity. Ongoing clinical trials for tyrosine kinase inhibitors involving TRK proteins are outlined in Table 2, and it will be an important task to examine the anti-cancer effects of these compounds.

Entrectinib (RXDX-101 or NMS-E628), is a potent, selective, ATP-competitive inhibitor of the tyrosine kinases TRKA/B/C, c-ros oncogene 1 (ROS1), and anaplastic lymphoma kinase (ALK) that is currently being investigated in multiple phase II studies [1,5]. The results from phase I trials with Entrectinib, namely ALKA-372-001 and STARTRK-1, have been promising and exciting. Patients enrolled in these trials presented a variety of tumors, including breast, renal cell, ovarian, NSCLC, primary brain, and sarcomas [1]. Of the 119 patients that were enrolled, 60 presented a gene rearrangement of NTRK1/2/3, ROS1, or ALK. Point mutations, amplifications, or gene insertions or deletions were observed in 53 patients. Six patients were enrolled, with no known alteration of NTRK1/2/3, ROS1, or ALK. With one exception, no objective response was observed in the patient group without a known alteration of the tyrosine kinases [1]. 

From this initial phase I trial, a phase II-eligible population was selected based on the presence of a gene fusion involving NTRK1/2/3, ROS1, or ALK and no prior TKI therapy. Of these 25 patients, the three with NTRK1/2/3 rearranged solid tumors experienced an objective response rate of 100% and included NSCLC, colorectal, and glioneural tumors [1]. Patients with ROS-1 rearranged solid tumors demonstrated an objective response rate (ORR) of 86%, including two complete responses and melanoma, and NSCLC. In the remaining seven patients with ALK rearrangements, an ORR of 57% was achieved, and included NSCLC, renal cell carcinoma, and colorectal cancer. Although the tumor histology varied widely in this study, the median progression-free survival for patients harboring rearrangements in NTRK1/2/3, ROS1, and ALK is 19.0 months. While this median overall survival time point has not been reached, 89.4% of patients are surviving at 12 months [1]. 

Remarkably, Entrectinib has demonstrated the efficacy against CNS tumors, indicating the potential and ability for blood-brain barrier penetration [5]. Of the 25 phase II-eligible patients, eight had a primary or metastatic disease that involved the brain. CNS responses were observed in 63% of these patients. Of note, one patient with 15–20 brain metastases (SQSTM1-NTRK1 NSCLC) had achieved a complete intracranial response that has extended beyond 15 months [1]. Besides, its promising antitumor effects, Entrectinib appears to be well-tolerated in humans. Side effects were mild to moderate and reversible with dose interruption or reduction [1]. The responses observed with Entrectinib treatment demonstrate that NTRK rearrangements are clinically relevant drivers of oncogenic growth. Because of the unsurprising failure of Entrectinib in patients without a tyrosine kinase rearrangement, it is imperative that comprehensive molecular profiling platforms are performed to identify those that would benefit from tyrosine kinase directed therapy. 

Like Entrectinib, LOXO-101 (Larotrectinib) is a pan-TRK inhibitor that is capable of penetrating the BBB and is currently tested in multiple phase II clinical trials for tumors with evidence of TRK fusions. Specific neoplasms include glioblastoma, NSCLC, colorectal cancers, melanoma, pancreatic, and ovarian, among others (Table 2). Pharmacokinetically, LOXO-101 has advantageous properties, including good systemic exposure following oral dosing and the 98% inhibition of TRK receptors at peak concentrations [90]. LOXO-101 showed early promise as an anti-neoplastic therapy in 2015 in a study enrolling six patients with NTRK fusions. In each case, tumors were pathologically advanced and previously pre-treated. All of the patients had confirmed partial responses to LOXO-101 within four months, and one patient with a soft-tissue sarcoma had a near complete response after eight months [91]. Importantly, the responses have proven to be durable and all of the patients are continuing therapy. LOXO-101 also presents a well-tolerated toxicity profile with fatigue, dizziness, and anemia among the most common side effects. As a follow-up to these promising results, LOXO-101 is currently being investigated in a multicenter phase II basket trial for patients with any NTRK-fusion-expressing solid tumor. Drilon et al. recently reported results of a phase I/II clinical trial on larotrectinib/LOXO-101 in children and adults showing its promising use for not only adults, but also children [92]. Because it has proven to be ineffective in patients lacking NTRK alterations, LOXO-101’s mechanism appears to be inhibition of genetically rearranged NTRK, although the exact mechanism is yet to be elucidated [91].

Cabozantinib (XL184, Cometriq) is an orally bioavailable small molecule inhibitor of c-Met, RET, ROS1, Alk, VEGFR2, and TRK receptors with approved uses for the treatment of metastatic medullary thyroid cancer and prostate cancer. In 2016, Cabozantinib was approved as an anti-angiogenic therapy for patients with advanced renal cell carcinoma with previous treatment, demonstrating significant improvements in response rates, progression-free survival, and overall survival [93,94]. While its use clinically is overwhelmingly used for renal cell carcinoma and metastatic medullary thyroid cancer, data is limited on its efficacy against NTRK fusions. However, with growing interest in TRK as a chemotherapy target, Cabozantinib is currently under investigations for patients harboring NTRK1 gene fusions in advanced NSCLC. While promising data has propelled Cabozantinib into Phase II trials, its reported use for NTRK1 fusions is limited. Because Cabozantinib has a dual capacity to inhibit angiogenesis and target gene fusions, the impact of this drug to cancer treatment may be substantial. Multiple phase I and II trials are currently underway to evaluate the use for cabozantinib in both brain metastases of various tumors, as well as high-grade gliomas [95]. While data is still limited in describing its use for CNS tumors, its demonstrated blood-brain barrier penetrance may prove to be effective for this site. 

Merestinib, is an inhibitor of c-Met, TEK, ROS1, and TRK1/2/3 being investigated in several phase II trials for advanced biliary tract cancer, NSCLC, and other various solid tumors. As of late September 2017, merestinib began a phase 1b trial for the treatment of bone metastases in breast cancer patients in combination with standard breast cancer therapies. Like other investigations of TRK inhibitors, merestinib exhibited early promise as a tyrosine kinase inhibitor in multiple in vivo cancer models. Besides its clinical trial recruitment for biliary tract cancer and NSCLC, merestinib has also shown potential for treating AML [96]. Currently, merestinib, in combination with the tyrosine kinase inhibitor LY2874455, is recruiting for a Phase II clinical trial for patients with relapsed or refractory AML. While data on the contribution of NTRK fusions in AML is rare, enhanced detection methods for gene fusions, along with increased exposure of TRK proteins as contributors to oncogenesis, may allow for TRK inhibitors to gain acceptance as a therapy for this cancer. 

TSR-011 is an orally bioavailable inhibitor of ALK and TRK receptor tyrosine kinases. Like TRK kinases, ALK gene rearrangements and dysregulated signaling are associated with a growing number of neoplasms. Promising in vitro and in vivo data also suggests a major role in suppressing the oncogenic growth in NSCLC [97]. In early 2014, TSR-011 began a phase I/II open-label, dose-escalation trial (NCT02048488) in patients with advanced solid tumors and lymphomas expressing altered or enhanced TRK or ALK activity [98]. Similar to LOXO-101 and Entrectinib, TSR-011 has had remarkable success in early clinical trials. In a phase I trial, TSR-011 treatment in patients presenting rearranged ALK or NTRK genes had an objective response rate of 100% in 46 patients [98,99].

DS-6051b is an orally active inhibitor of ROS and TRK tyrosine kinases that is currently in a Phase I, first-in-human clinical trial (NCT02279433). This study will also investigate the pharmacokinetic profile and the preliminary efficacy in humans, and provide dosing guidelines for subsequent clinical trials. Prerequisites for enrolling in this trial include advanced solid tumors containing ROS1 or NTRK gene rearrangements or advanced solid tumors causing tumor-induced pain. 

MGCD516 (Mirati Therapeutics Inc., San Diego, CA, USA) is another multi-kinase inhibitor in a phase 1/1b clinical trial (NCT02219711) for patients with advanced cancers expressing rearranged MET, RET, AXL, NTRK1, or NTRK3 genes. MGCD516 is also predicted to be effective for treating tumors with activating mutations or amplifications of MET, NTRK2, NTRK3, or DDR2. Regarding tumor type, NSCLC, clear cell renal cell carcinoma, metastatic prostate cancer, and head and neck squamous cell carcinoma are anticipated to be the major histologies [100]. As of 2017, this trial is currently enrolling patients and will hopefully produce exciting response rates for these patients. 

PLX7486 (Plexxikon, Berkeley, CA, USA) is currently being investigated as a treatment for any solid tumor, demonstrating an activating NTRK mutation (NCT01804530). This trial encompasses PLX7486 as a single-agent treatment as well as in combination. Because TRKA signaling is implicated in pain, this trial is also being evaluated as a modulator for cancer-related pain [11]. Similar to MGCD516, DCC-2701, and MGCD516 are multi-tyrosine kinase inhibitor with in vitro activity against TRKA and TRKB. DCC-2701 and MGCD516 have both recently entered phase I clinical trials for solid tumors expressing gene fusions, and will hopefully generate promising and durable results.

## 7. Resistance to TRK Inhibitors

Because TRK inhibitors are fairly new to clinical development, information regarding the acquired clinical resistance is scarce. Point mutations in the kinase domain are predicted to affect sensitivity, but few have emerged as being clinically relevant. However, a recent study has documented genetic alterations affecting Entrectinib sensitivity in a patient carrying a LMNA-NTRK1 fusion. To characterize this mechanism of resistance, circulating tumor DNA (ctDNA) was transplanted into immunodeficient mice to generate a patient-derived xenograft [101]. The xenograft was then administered the same treatment until resistance emerged, and two novel NTRK protein point mutations were observed. In the kinase domain of the protein, G595R and G667C were observed to emerge in these mice [101]. While these point mutations arose in a patient-derived xenograft following entrectinib administration, this preclinical observation of acquired resistance may emerge as clinically relevant, as clinical trials progress and as identification of gene fusions develops. A recent study showed G667C mutation-expressing entrectinib-resistant brain-metastatic colon cancer of cells responded to another NTRK1 inhibitor foretinib [102]. Combining existing cancer treatments, such as foretinib, doxorubicin, cisplatin, and irinotecan with TRK inhibitors could potentially overcome entrectinib resistance, although clinical data regarding combination treatments is still lacking.

## 8. Conclusions

While TRK proteins serve a vital role in CNS development and survival, the aberrant signaling of TRK proteins is implicated in a variety of malignancies. The identification of NTRK fusions and mutations as oncogenic contributors has revealed opportunities for therapeutic intervention. The early clinical success of molecules, such as LOXO-101, Entrectinib, and Cabozantinib have shown that the therapeutic target can potentially more important than the tumor organ when selecting a treatment. As technologies allow for more gene rearrangement data, therapies can be better planned to target the oncogenic contributor. Although TRK inhibitor research is in its early stage of clinical evaluation, the future appears promising for patients with TRK mutations and the field of molecularly targeted therapies.

## Figures and Tables

**Table 1:** Clinically identified and reported NTRK family gene fusions and associated cancers.

NTRK Gene	Fusion Protein Partner	Cancer Type	Reference
**NTRK1**	**ARHGEF2**	Glioblastoma	Zheng et al., (2014) [44]
**NTRK1**	**BCAN**	Glioblastoma	Kim et al., (2014) [36] Frattini et al., (2013) [40]
**NTRK1**	**CD74**	Lung adenocarcinoma	Vaishnavi et al., (2013) [45]
**NTRK1**	**CHTOP**	Glioblastoma	Zheng et al., (2014) [44]
**NTRK1**	**LMNA**	AYA sarcoma	Doebele et al., (2015) [46]
		Colorectal	Sartore-Bianchi et al., (2016) [2]
		Congenital infantile fibrosarcoma	Wong et al., (2015) [47]
		Spitzoid melanomas	Wiesner et al., (2015) [48]
**NTRK1**	**MPRIP**	Lung adenocarcinoma	Vaishnavi et al., (2013) [45]
**NTRK1**	**NFASC**	Glioblastoma	Kim et al., (2014) [36] Frattini et al., (2013) [40]
**NTRK1**	**PPL**	Thyroid carcinoma	Farago et al., (2015) [49]
**NTRK1**	**RABGAP1L**	Intrahepatic cholangiocellular carcinoma	Ross et al., (2014) [50]
**NTRK1**	**RFWD2**	Large cell neuroendocrine tumor	Fernandez-Cuesta et al., (2014) [51]
**NTRK1**	**SQSTM1**	Lung adenocarcinoma	Farago et al., (2015) [49]
**NTRK1**	**TFG**	Papillary thyroid carcinoma	Beimfohr et al., (1999) [52] Greco et al., (2010) [53]
**NTRK1**	**TP53**	Spitzoid melanomas	Wiesner et al., (2014) [48]
**NTRK1**	**TPM3**	Colorectal cancer	Martin-Zanca et al., (1986) [37] Creancier et al., (2015) [54] Ardini et al., (2014) [55]
		Glioblastoma	Wu et al., (2014) [41]
		Papillary thyroid carcinoma	Bongarzone et al., (1989) [38] Beimfohr et al., (1999) [52] Butti et al., (1995) [56]
**NTRK1**	**TPR**	Papillary thyroid carcinoma	Greco et al., 1992, 1997 [57,58]
		Colorectal cancer	Creancier et al., 2015 [54]
**NTRK1**	**SCYL3**	Colorectal cancer	Milione et al., 2017 [43]
**NTRK2**	**AFAP1**	Low-grade glioma	Stransky et al., (2014) [59]
**NTRK2**	**AGBL4**	Glioblastoma	Wu et al., (2014) [41]
**NTRK2**	**NACC2**	Pilocytic astrocytoma	Jones et al., (2013) [60]
**NTRK2**	**PAN3**	Head and neck squamous cell carcinoma	Wu et al., (2014) [41] Stransky et al., (2014) [59]
**NTRK2**	**QKI**	Pilocytic astrocytoma	Jones et al., (2013) [60]
**NTRK2**	**TRIM24**	Lung adenocarcinomas	Wu et al., (2014) [41] Stransky et al., (2014) [59]
**NTRK2**	**VCL**	Glioblastoma	Wu et al., (2014) [41]
**NTRK3**	**BTBD1**	Glioblastoma	Wu et al., (2014) [41]
**NTRK3**	**ETV6**	Acute myelogenous leukemia	Kralik et al., (2011) [24] Eguchi et al., (1999) [61] Knezevich et al., (1998a) [62]
		Congenital fibrosarcoma	Knezevich et al., (1998b) [63]
		Congenital mesoblastic nephroma	Knezevich et al., (1998a) [62] Rubin et al., (1998) [64] Watanabe et al., (2002) [65]
		Colorectal cancer	Hechtman et al., (2015) [66]
		Ductal carcinoma	Makretsov et al., (2004) [67] Arce et al., (2005) [68] Pinto et al., (2014) [69]
		Fibrosarcoma	Morerio et al., (2004) [70] Punnett et al., (2000) [71]
		Gastrointestinal stromal carcinoma	Brenca et al., (2015) [72]
		Glioblastoma	Wu et al., (2014) [41]
		Mammary analogue secretory carcinoma	Tognon et al., (2002) [39] Skalova et al., (2016) [73] Ito et al., (2015) [74] Del Castillo (2015) [75]
		Papillary thyroid carcinoma	Leeman-Neill et al., (2014) [76]

**Table 2:** Ongoing clinical trials for patients with NTRK fusions or altered activity.

NCT Identifier	Drug	Phase	Cancer Type Indication
NCT03213704	LOXO-101 (Larotrectinib)	II	Advanced malignant solid neoplasm, malignant glioma, recurrent central nervous system neoplasms, childhood neoplasms (ependymoma, malignant germ cell, medulloblastoma, Non-Hodgkin Lymphoma, Rhabdomyosarcoma, soft Tissue Sarcoma) Ewing Sarcoma, glioma, hepatoblastoma, Langerhans cell histiocytosis, neuroblastoma, osteosarcoma, peripheral primitive neuroectodermal tumor, refractory Central Nervous System Neoplasms, Wilms tumors
NCT02637687	LOXO-101 (Larotrectinib)	II	Neoplasms and central nervous system neoplasms
NCT02576431	LOXO-101 (Larotrectinib)	II	NSCLC, Thyroid, Sarcoma, Colorectal, Salivary Gland, Biliary Tract, primary brain, ductal breast, melanoma, solid tumors, bile duct astrocytoma, head and neck, squamous cell, pontine glioma, pancreatic, ovarian, renal, cholangiocarcinoma, bronchogenic, lung, thoracic cavity, nevi and melanomas
NCT02122913	LOXO-101 (Larotrectinib)	I	Adult solid tumors
NCT02568267	Entrectinib	II	Breast, cholangiocarcinoma, colorectal, head and Neck, large-cell anaplastic lymphoma, melanoma, neuroendocrine, NSCLC, ovarian, pancreatic, papillary thyroid, primary brain tumors. renal cell carcinoma, sarcomas, salivary gland, other adult solid tumor
NCT02097810	Entrectinib	II	Locally advanced solid tumors and metastatic solid tumors
NCT02650401	Entrectinib	II	Solid tumors, CNS tumors, and neuroblastoma
NCT01639508	Cabozantinib	II	NSCLC
NCT02920996	Merestinib	II	Carcinoma, NSCLC, and other solid tumors
NCT02048488	TSR-011	I/II	Solid tumors, lymphomas
NCT02279433	DS-6051b	I	Solid tumors, lymphomas
NCT02219711	MGCD516	I	Advanced cancers
NCT01804530	PLX7486	I	Solid tumors and tenosynovial giant cell tumors
NCT02228811	DCC-2701	I	Locally advanced tumors and metastatic solid tumors
